# Commentary: COVID-19 Pandemic Response and Research in Africa: Global Health Hypocrisy at Work?

**DOI:** 10.3389/fpubh.2021.790996

**Published:** 2022-04-04

**Authors:** Claude Ngwayu Nkfusai, Caroline Ekoko Subi, Epo Gaelle Larissa, Paschal Kum Awah, Hubert Amu, Claudine Akondeng, Olivia Ngou, Luchuo Engelbert Bain

**Affiliations:** ^1^Impact Santé Afrique (ISA), Yaoundé, Cameroon; ^2^Department of Public Health, School of Nursing and Public Health, University of Kwa-Zulu Natal, Durban, South Africa; ^3^Local Youth Corner (LOYOC), Yaoundé, Cameroon; ^4^Service de Pediatrie, Hopital Antoine Beclere, Clamart, France; ^5^Department of Anthropology, Faculty of Arts, Letters and Social Sciences, University of Yaoundé I, Yaoundé, Cameroon; ^6^Department of Population and Behavioural Sciences, School of Public Health, University of Health and Allied Sciences, Hohoe, Ghana; ^7^Cameroon National Association for Family Welfare (CAMNAFAW), Yaoundé, Cameroon; ^8^College of Social Science, International Institute of Rural Health, University of Lincoln, Lincoln, United Kingdom

**Keywords:** COVID-19 pandemic, response, research, Africa: global health, hypocrisy

## Summary

- The projected worst-case scenario with no interventions was that Africa could see 3.3 million deaths and 1.2 billion infections as of mid-November 2020.
- The place of African traditional medicine in this pandemic has been central but yet underreported and almost not carefully systematically researched as expected.
- The reality presented by lack of interest to carefully investigate the failed predictions of doom for the African continent regarding the COVID-19 pandemic showcases global health hypocrisy, and the fact that global health justice in research is a meaningless concept.
- Indeed, no enthusiasm has been shown by the international research community to understand empirically the reasons for this “resistance.”
- Imported models insensitive to the sociocultural and economic realities are needed no more. Africa and the international community stand to gain with a deeper understanding of why the expected doom never came. This might be useful to build on the gains and better prepare for future pandemics.

The most prominent of the projections was that of the United Nations Economic Commission for Africa (UNECA) on April 2020 which projected that Africa could see about 300,000 deaths from COVID-19 during the year even under the best-case scenarios (1). The projected worst-case scenario with no interventions was that Africa could see 3.3 million deaths and 1.2 billion infections. These projections by UNECA were linked to the fact that the continent has one of the world's weakest and fragile health systems with dilapidated healthcare infrastructure and the number of doctors per capita is among the lowest in the world (1). Contrary to the projections of doom for Africa, a World Health Organization (WHO) report released on the 21st and 26th May 2021 showed that Africa remains the continent with the lowest number of confirmed cases of 3,457,590 compared to 66,414,286 in the Americas, 53,901,476 in Europe, 30,781,898 in South East Asia and 9,955,811 in Eastern Mediterranean. Africa is still the continent with the lowest number of COVID-19 related deaths of 86,220 against 1,625,371 in the Americas, 1,140,008 in Europe, 385,673 in South East Asia, and 199,581 Eastern Mediterranean (2). Countries rated as highly prepared on the Global Health Security Index (3) did not perform as well as expected with COVID-19. For instance, the United States which ranks 1st did not do so well going by the COVID-19 infected persons and death rates (3). Going by the relatively low COVID-19 related reported deaths, Africa's fight against the pandemic can be considered as successful.

Adamset al. (4) have hypothesized some explanations for the reported low COVID-19 incidence and deaths in Africa around six main points: demographic structure, lack of long-term care facilities, cross protecting from other previously circulating corona viruses, underreporting, genetic factors, and public health mitigation strategies.

## Early Interventions

Unlike the rest of the world, as soon as COVID-19 was declared by WHO as a global concern, African states became tactical in their response as they initiated strategies to tackle the virus and every country went on red alert. Strict measures including travel restrictions, lockdown, and cancellation/limitation on the number of persons at public gatherings were imposed earlier. The immediate shutdown of their respective economies was highly criticized by the rest of the world, and had a lot of negative effects on their already struggling economies but, slowed the spread of the virus (4). Rwanda, for instance, took one of the most aggressive measures as it canceled all flights from China on January 31, 2020 and later suspended all flights, closed its borders, and told residents to remain indoors. The rest of the world, however, took a longer time to shut down their economies and by the time they did, the virus had already gained dominance (4).

## Solidarity Between African States

Africans have always been united in the fight against epidemics and pandemics and this has been the same for COVID-19 (4, 5). Since the emergence of the virus, African leaders have held several virtual meetings to discuss the continent's response to the pandemic. The private sector in many African countries has donated generously to COVID-19 solidarity funds and the African Center for Disease Control and Prevention (Africa CDC) has shown leadership in supporting the continent's response.

## Experience From Past Pandemics and Epidemics

The continent had learned her bitter lessons from previous pandemics such as Ebola, cholera, influenza and HIV/AIDS. As such African governments took aggressive preventive measures before they even had their first confirmed cases. Her response to these pandemics and epidemics had taught her to invest in more resilient health systems as well as put in place strong and effective surveillance mechanisms and coordination reflexes to cope with future epidemics and pandemics (4). In the aftermath of the Ebola crisis, the World Bank launched the Regional Disease Surveillance Systems Enhancement (REDISSE) Project to strengthen health systems and support effective disease surveillance in 16 West and Central African countries. The first laboratory on the continent to be accredited by the WHO for the testing of COVID-19 cases was the Institute Pasteur in Dakar. The team of the institute started preparing in January 2020 before even the first cases were recorded in Africa as the laboratory was used in handling previous disease outbreaks (6). Also, countries such as Nigeria and Cameroon used lessons learned from the Ebola epidemic to quickly set isolation clinics to treat solely COVID-19 patients (4).

## The Use of Traditional Medicines

While the rest of the world was waiting for the development of some clinically tested treatment, Africans did not wait for their government to propose biomedical treatment developed elsewhere. Traditional medicine is the foundation of healthcare in Africa. It is comprised largely of herbal medications. Also making up the majority of alternative medicine, herbal medications constitute the backbone of many African countries with poorly resourced orthodox healthcare systems. The use of traditional herbal medicine, thus, increased greatly in the midst of the COVID-19 pandemic as many more people turned to herbs believing it treats and prevents many diseases. There has been a lot of misinterpretation regarding the use of herbal plants all across Africa. With no known cure for COVID-19, Africa took the lead with herbal medicine, notably in Madagascar and Cameroon (7, 8). The president of Madagascar, Andry Rajoelina, for instance, approved a locally manufactured herbal tonic called COVID Organics (CVO) for the prevention and treatment of COVID-19. Made from the Artemisia plant that contains antimalarial properties, the president hailed COVID Organics as the panacea for COVID-19 in April 2020. Many other African countries including the Republic of the Congo, Equatorial Guinea, Tanzania, and Guinea Bissau went ahead to order the herbal medicine in a show of solidarity. In Cameroon, a Catholic Archbishop Kleda announced the successful treatment of COVID-19 patients with a herbal mixture which principally targets respiratory problems associated with the disease. The mixture is given free of charge in catholic health centers after apparent presentation of positive results. The ministry of public health in Cameroon has recently authorized the commercialization of 4 traditional medicines as adjuvant therapies against COVID-19 (8). African traditional medicine needs intensive research investments. This has neither been prioritized by African governments themselves, nor the “international community.” Generating evidence with robust research (for instance Randomized Controlled Trials) to showcase the effectiveness of these medicines will constitute a strong advocacy tool to enhance ownership, trust, and respect. Though highly criticized, most African countries adopted the Chloroquine—Azithromycine protocol in managing COVID-19 patients. Findings from clinical trials elsewhere have almost been conclusive on the fact that this protocol failed to neither improve health outcomes nor reduce deaths (9, 10). However, no robust clinical trials have systematically investigated patient outcomes on this protocol in Africa. In a recent prediction model study, countries rated as better prepared and having more resilient health systems in Sub–Saharan Africa were worst affected by the disease (11). It is known that social distancing measures and protective measures like wearing of face masks are poorly respected in most populated African states. The current vaccination trends remain low in SSA. Common sense warrants death and hospitalization rates to be highest in this region of the world, despite the reported circulation of the delta variant of the virus. The resistance to this doom is worth investigating.

## The Research Imperative

The reality presented by the failed predictions of doom for the African continent regarding the COVID-19 pandemic showcase global health hypocrisy and the fact that global health justice in research is a meaningless concept (12). A robust research agenda with accompanying funding should have been put in place to understand this unexpected resistance to COVID-19. In a modeling study by Haug et al. intrusive and drastic interventions like national lockdowns are not always more effective than less disruptive interventions (13). Indeed, health policies put in place in high income countries (school lockdowns and travel restrictions for instance) were non-uniform within and across respective states. It is impossible today to state with precision which possibility or implementation actually worked (14). The hypothesis put forward by in this commentary by Adams and colleagues (4) are proof of the fact that understanding the less than expected infection and death rates from COVID-19 in Africa should constitute a research priority. The demographic structure and health system weaknesses of the region were known before the predictions of doom were made. Braving the odds (resisting) therefore warrants evidence-based explanations and should constitute a priority for learning for Africa and the rest of the world. Not investing adequately in research to understand why Africa stood strong in the face of this pandemic will be a glaring example of global health injustice in research. Africa undoubtedly needs to work extremely hard to come up with its own health system priority setting and strengthening models (5). Imported models insensitive to the sociocultural and economic realities are not very much needed. The fact that early predictions failed is no excuse to go comfortable with existing measures put in place.

## Conclusion

Since its emergence in December 2019 in China, COVID-19 has ravaged countries across the globe with disturbingly high mortality and morbidity rates. While there were predictions of Africa bearing the heaviest COVID-19 related morbidity and mortality globally, this never happened. Explanations for this resistance have mainly been on a hypothetical frame. Ignoring this “African Resistance” against COVID-19 as a research priority is proof of astute injustice in global health research. Africa and the international community stand to gain with a deeper understanding of why the expected doom never came. This might be useful in preparing for future pandemics. COVID-19 is still here. Staying focused on prevention and avoiding community transmission, testing and contact tracing, case management, stepping up vaccination, and investing in research are priority action areas to successfully fight the pandemic.

## Author Contributions

All authors listed have made a substantial, direct, and intellectual contribution to the work and approved it for publication.

## Conflict of Interest

The authors declare that the research was conducted in the absence of any commercial or financial relationships that could be construed as a potential conflict of interest.

## Publisher's Note

All claims expressed in this article are solely those of the authors and do not necessarily represent those of their affiliated organizations, or those of the publisher, the editors and the reviewers. Any product that may be evaluated in this article, or claim that may be made by its manufacturer, is not guaranteed or endorsed by the publisher.
